# Knee Pain during Strength Training Shortly following Fast-Track Total Knee Arthroplasty: A Cross-Sectional Study

**DOI:** 10.1371/journal.pone.0091107

**Published:** 2014-03-10

**Authors:** Thomas Bandholm, Kristian Thorborg, Troels Haxholdt Lunn, Henrik Kehlet, Thomas Linding Jakobsen

**Affiliations:** 1 Physical Medicine & Rehabilitation Research – Copenhagen (PMR-C), Department of Physical Therapy, Hvidovre Hospital, University of Copenhagen, Copenhagen, Denmark; 2 Clinical Research Centre, Hvidovre Hospital, University of Copenhagen, Copenhagen, Denmark; 3 Department of Orthopedic Surgery, Hvidovre Hospital, University of Copenhagen, Copenhagen, Denmark; 4 Arthroscopic Center Amager, Amager Hospital, University of Copenhagen, Copenhagen, Denmark; 5 Department of Anaesthesiology, Hvidovre Hospital, University of Copenhagen, Copenhagen, Denmark; 6 Lundbeckfoundation Centre for Fast-track Hip and Knee Surgery, Hvidovre Hospital, University of Copenhagen, Copenhagen, Denmark; 7 Section for Surgical Pathophysiology, Rigshospitalet, University of Copenhagen, Copenhagen, Denmark; The University of Tokyo Hospital, Japan

## Abstract

**Background:**

Loading and contraction failure (muscular exhaustion) are strength training variables known to influence neural activation of the exercising muscle in healthy subjects, which may help reduce neural inhibition of the quadriceps muscle following total knee arthroplasty (TKA). It is unknown how these exercise variables influence knee pain after TKA.

**Objective:**

To investigate the effect of loading and contraction failure on knee pain during strength training, shortly following TKA.

**Design:**

Cross-sectional study.

**Setting:**

Consecutive sample of patients from the Copenhagen area, Denmark, receiving a TKA, between November 2012 and April 2013.

**Participants:**

Seventeen patients, no more than 3 weeks after their TKA. Main outcome measures: In a randomized order, the patients performed 1 set of 4 standardized knee extensions, using relative loads of 8, 14, and 20 repetition maximum (RM), and ended with 1 single set to contraction failure (14 RM load). The individual loadings (kilograms) were determined during a familiarization session >72 hours prior. The patients rated their knee pain during each repetition, using a numerical rating scale (0–10).

**Results:**

Two patients were lost to follow up. Knee pain increased with increasing load (20 RM: 3.1±2.0 points, 14 RM: 3.5±1.8 points, 8 RM: 4.3±2.5 points, P = 0.006), and repetitions to contraction failure (10% failure: 3.2±1.9 points, 100% failure: 5.4±1.6 points, P<0.001). Resting knee pain 60 seconds after the final repetition (2.7±2.4 points) was not different from that recorded before strength training (2.7±1.8 points, P = 0.88).

**Conclusion:**

Both loading and repetitions performed to contraction failure during knee- extension strength-training, increased post-operative knee pain during strength training implemented shortly following TKA. However, only the increase in pain during repetitions to contraction failure exceeded that defined as clinically relevant, and was very short-lived.

**Trial Registration:**

ClinicalTrials.gov NCT01729520

## Introduction

Total knee arthroplasty (TKA) is a frequently-used procedure to relieve pain and improve function in advanced osteoarthritis of the knee joint [1]. Despite the fact that the patients enter a fast-track or enhanced peri-operative recovery program [2], they loose, on average, 80% knee-extension strength over the 2–3 days of their hospitalization [3]. It is the most pronounced acute loss of knee-extension strength of any cohort of people who have had knee surgery [4], and is caused by failure of the central nervous system (CNS) to activate the quadriceps muscle [5], [6] – known as arthrogenic quadriceps muscle inhibition [7].

The mechanisms underlying arthrogenic quadriceps muscle inhibition are thought to be altered afferent signalling from the operated knee joint due to swelling, inflammation, and damage to joint afferents, which affects the CNS by changing the excitability of multiple spinal and supraspinal pathways [7]. Ultimately, CNS activation of the quadriceps muscle is reduced, whereby the ability to produce knee-extension force for functional tasks is also reduced. Hence, rehabilitation modalities that are known to increase CNS activation of the quadriceps muscle are indicated after TKA. However, fear of symptom exacerbation such as increased postoperative knee pain has typically precluded such intense therapeutic exercise [8], [9], likely because maximal dynamic quadriceps contractions will also increase the load on the operated knee joint. These precautions may be well founded, but scientific data are needed to address the concerns. Moreover, when (or if) prescribing quadriceps strengthening exercises following TKA, it is important to know how different strength training variables, such as loading and repetitions performed until contraction failure, influence postoperative knee pain.

When undertaking strength training to increase CNS activation of the targeted muscle, heavy loading and/or contractions performed to or near contraction failure (muscular exhaustion) are emphasized [10]. That is, CNS activation of the contracting muscle increases as contraction failure is approaching in healthy subjects [11], and strength training with heavy loading results in greater CNS activation of the exercising muscle compared low loading in healthy subjects [12]. Hence, both heavy loading and repetitions performed to contraction failure seem logic choices when strength training the quadriceps muscle to reduce arthrogenic inhibition following TKA. Indeed, quadriceps contractions have been shown to reduce artificially-induced arthrogenic quadriceps muscle inhibition in an experimental human model of knee swelling [13].

The objective of this study was to investigate how loading and repetitions to contraction failure influence knee joint pain during knee-extension strength training with the operated leg shortly following TKA. We hypothesized that knee pain would increase with increasing loading and contraction failure.

## Methods

### Ethics statement

All patients were provided written information about the procedures of the study, and written informed consent was obtained in strict accordance with the Declaration of Helsinki, using the template provided by The Committees on Biomedical Research Ethics for the Capital Region of Denmark. The Committees on Biomedical Research Ethics for the Capital Region of Denmark decided that a full study protocol was not needed for approval, as the study was part of existing clinical practice. Hence, the study was approved by The Committees on Biomedical Research Ethics for the Capital Region of Denmark (ref: H-4-2011-fsp (108)), based on a review of a brief study protocol (please see Supporting Information S1). Hereafter, study details were added, and these were listed in the registration of the trial (NCTO1729520, http://clinicaltrials.gov/ct2/show/NCT01729520).

### Design

Patients, having received a primary unilateral TKA less than three weeks earlier, reported knee pain while undertaking one strength training session of the quadriceps muscle in the operated leg, using relative loads of 20, 14, and 8 repetition maximum (RM) in a randomized order. They ended the session with a single set performed to contraction failure, using a relative load of 14 RM. The absolute loads (kilograms lifted) corresponding to 20, 14, and 8 RM, were determined at a familiarization session no less than 72 hours earlier to avoid differences in the number of trials required to determine the absolute loads at the experimental session. The reporting of the study follows the recommendations of The Strengthening the Reporting of Observational Studies in Epidemiology (STROBE, [please see Supporting Information, S2]) [14]. The present study reports the findings from the knee-extension exercise in the approved protocol (please see Supporting Information S1). A similar study investigating the effect of loading and repetitions performed until contraction failure outlined in the approved protocol has not been conducted yet.

### Participants

Between November 2012 and April 2013, patients referred to outpatient rehabilitation at a single rehabilitation institution in the area of Copenhagen after a TKA, were included by consecutive sampling. The inclusion criteria were: primary unilateral TKA 1 to 2 weeks earlier, age between 18 and 80 years, ability to speak and understand Danish. The exclusion criteria were: other diseases, such as rheumatoid arthritis, polyneuropathy, or extremity peripheral paresis, alcohol or drug abuse, and severely restricted range of knee joint motion (less than from 90 to 40 degrees of flexion).

### Procedures

#### Familiarization session

**Figure 1:**
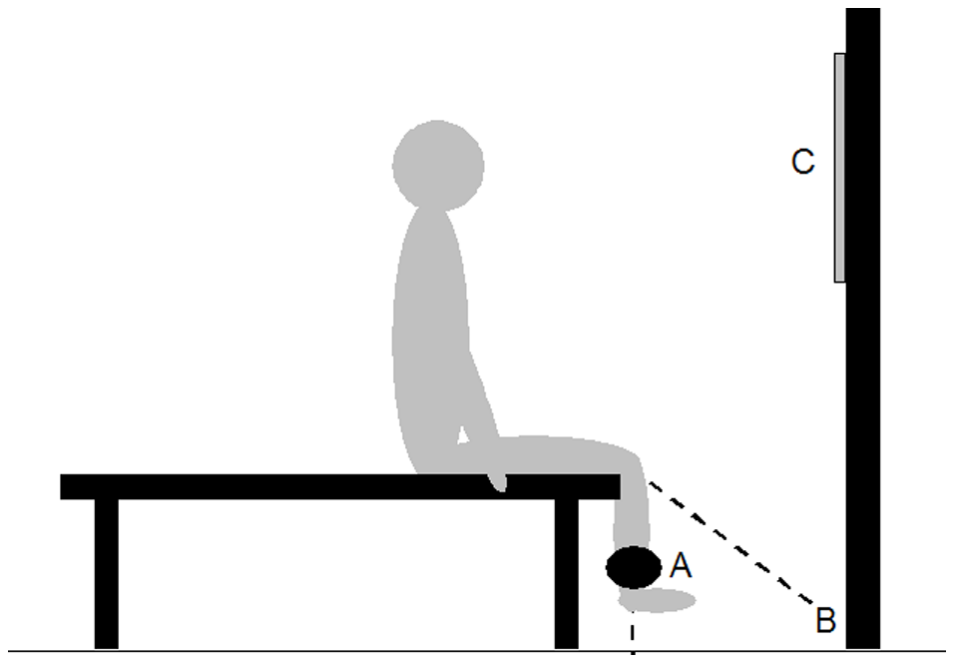
Experimental arrangement. The patients sat at the end of an examination couch with weight cuffs attached to the ankle of the operated leg (A). They performed controlled knee extensions in a standardized range of motion indicated on the wall (B), while facing a numerical rating scale placed in front of them (C).

The patients were seated at the end of an examination couch with hip and knee angles of 90 degrees, respectively, and allowed to place their hands on the sides of the couch for upper-body fixation (Figure 1). They were instructed to maintain this posture during the loaded knee extensions. They then performed controlled knee extensions with the operated leg, to establish the load (kilograms) that could be lifted exactly 8 times using proper technique, corresponding to 8 RM. Lines drawn at the wall next to the couch ensured a 50 degrees controlled range of motion. Average contraction velocity was controlled by an audio file, ensuring a 3-second concentric contraction, a 3 second eccentric contraction, and 1 second isometric pauses at the ends of the concentric and eccentric phases, respectively. The 8 RM load was established in all patients. Subsequently, the 8 RM load was used to estimate 1 RM loading in all patients, based on the Brzycki equation [15]. From the 1 RM load, 14 and 20 RM loads were estimated as 65% and 50% of the 1 RM load, respectively. Having established the 8 RM load, the patients were familiarized with rating their knee pain during the loaded knee extensions. It was emphasized that the pain rating should apply to the operated knee joint and not the anterior thigh.

#### Experimental session

Load experiment: Using the same experimental setup as for the familiarization session, the patients performed 1 set of 4 repetitions for each of the relative loads: 20, 14, and 8 RM, respectively. The repetitions were standardized in the same way as described for the familiarization session. The absolute loads (kilograms) were attached as weight cuffs to the ankle of the operated leg and covered by a cloth, to ensure blinding of the loads. The weight cuffs weighed from 100 grams (lightest) to 5 kilograms (heaviest), and allowed the loads to be adjusted at the 100 g level.

Failure experiment: At the end of the experimental protocol, the patients performed a final set of contractions until contraction failure, using a relative load of 14 RM.

### Outcome measures

#### Primary outcome

At the experimental session, the patients rated their knee pain in the operated leg during the concentric phase of every contraction, using a numerical rating scale with endpoints of 0 (no pain) and 10 (worst pain imaginable) [16]. A large piece of paper with a drawing of the numerical rating scale was placed in front of the patients, 1 meter away. One single assessor recorded all pain ratings, while being blinded to the loads. For the load experiment, a mean of the 4 ratings was made for each of the 3 relative loads to reflect average knee pain for each load, respectively, and used as the data points. For the failure experiment, the pain ratings were normalized by expressing them as deciles (10%, 20%, etc.). This was done, as some patients performed exactly 14 repetitions with the pre-determined 14 RM load, whereas others performed, for example 16 repetitions. These deciles were used as data points.

#### Secondary outcomes

A number of secondary outcomes were recorded to indicate if the experimental session acutely exacerbated postoperative symptoms.

Knee pain in the operated leg was registered at rest and during tasks of everyday living to serve as knee pain references for the pain recordings made during exercise at the experimental session. With the patients in the supine position and knees slightly flexed, knee pain at rest was registered before commencing the experimental protocol and 60 seconds after the termination of the failure experiment (end of the experimental protocol). The recordings made during tasks of everyday living were: pain after 10 meters of level ground walking, and pain after 60 seconds of quiet standing with equal weight bearing. As for the primary outcome, the numerical rating scale was used. One recording was made for all assessments and used as data points.

Knee joint circumference of the operated knee was measured immediately before the load experiment and 120 seconds after the failure experiment to indicate knee joint swelling. The measurements were made with the patients in the supine position, according to a procedure previously described [17]. Briefly, a non-elastic tape measure was snug above a small dot made with a pen 1 cm proximal to the base of the patella, and the joint circumference was measured to the nearest 0.1 cm. Two recordings were made, and the largest circumference was used as the data point.

Knee flexion range of motion was measured immediately before the load experiment and 180 seconds after the failure experiment to indicate if the experimental session exacerbated post-operative knee flexion range of motion in the operated leg. The measurements were made with the patients in the supine position, according to a procedure previously described [17]. Briefly, a goniometer with 30-cm movable arms was placed with the fulcrum visually aligned to the trans-epicondylar axis of the knee joint. The tester then flexed the knee to a point where the patients said “stop” because of discomfort or threshold of pain, or the tester could not manually pres the knee into further flexion. When the tester was satisfied with the alignment, the movable armspointing towards the greater trochanter and the lateral malleolus, respectively, the goniometer was removed and the value was read to the nearest degree. Two recordings were made, and the highest knee flexion range of motion was used as the data point.

### Statistical analyses

The sample size for the present study was calculated based on data from a pilot study, conducted after the approved protocol (Supporting Information, S1). During this process, the sample size was adjusted compared to that estimated in the approved protocol, and the new sample size was reported in the registration of the trial (NCT01729520). From the pilot study, the mean difference in knee pain between the lightest and heaviest load – the 20 and 8 RM loads – was 1.1 ± 1.4 (SD) points (load experiment) and 2.4 ± 1.1 points between the first and last decile −10 and 100% failure – (failure experiment). Hence, the load experiment determined the sample size, as the greatest sample was required for this experiment (10 patients). Given an average knee pain rating of approximately 3 points at 20 RM (load experiment) and 10% failure (failure experiment), respectively, the minimal clinically important difference (increase) was defined as 1.5 points. A standard of 80% power and type I error rate of 5% was used. As the sample size calculations were performed using underlying t-tests, and we did not know if the final data would demonstrate a normal distribution, we adjusted the sample size by multiplying the sample size with 1.5, resulting in a sample size of 15 patients [18]. To account for potential dropouts between the familiarization and experimental sessions, 17 patients were included.

All final data were examined for normal distribution using Shapiro-Wilk tests and Q-Q plots, and found to be normally distributed. Hence, parametric tests were applied, and data are presented as means ±1 SD. Repeated measures ANOVAs were used to examine main effects of load (load experiment) and contraction failure (failure experiment). Paired samples t-tests were used to examine the effect of the experimental protocol on resting pain, knee joint circumference, and knee flexion range of motion. The level of significance was set at less than 5% (P<0.05).

## Results

### Participants

The number of potentially eligible patients was 30, as this was the total number of patients referred to outpatient rehabilitation after TKA at the participating rehabilitation site during the study period. All were examined for eligibility. Of these, 13 were excluded: 4 did not wish to participate, 1 due to re-operation of the knee before the start of outpatient therapy, 1 due to postoperative infection of the knee, 3 did not speak or understand Danish, 2 due to limited range of knee joint motion, 1 due to outpatient therapy being the second round of therapy (too late after surgery), and 1 due to therapy being home-based. The number of patients confirmed eligible was 17 and these were included. Two patients dropped out after the inclusion: 1 before the familiarization session due to illness at the day of the familiarization session, and 1 after the familiarization session due to illness (not study-related) at the day of the experimental session. The baseline characteristics of the final sample of 15 patients, who attended both sessions and had complete data sets, are shown in Table 1.

**Table 1:** Patient characteristics (n = 15).

Category	Result
Age, yrs	72.0±10.1
Women, number (%)	11 (73)
Height, cm	168.5±7.4
Body Mass, kg	86.1±13.5
Body Mass Index, kg/m^2^	30.2±3.2
Experimental session, days after surgery	12.5±4.7

Values are means ±1SD, unless otherwise indicated.

### Effect of load and contraction failure on knee pain (primary outcome)

**Figure 2:**
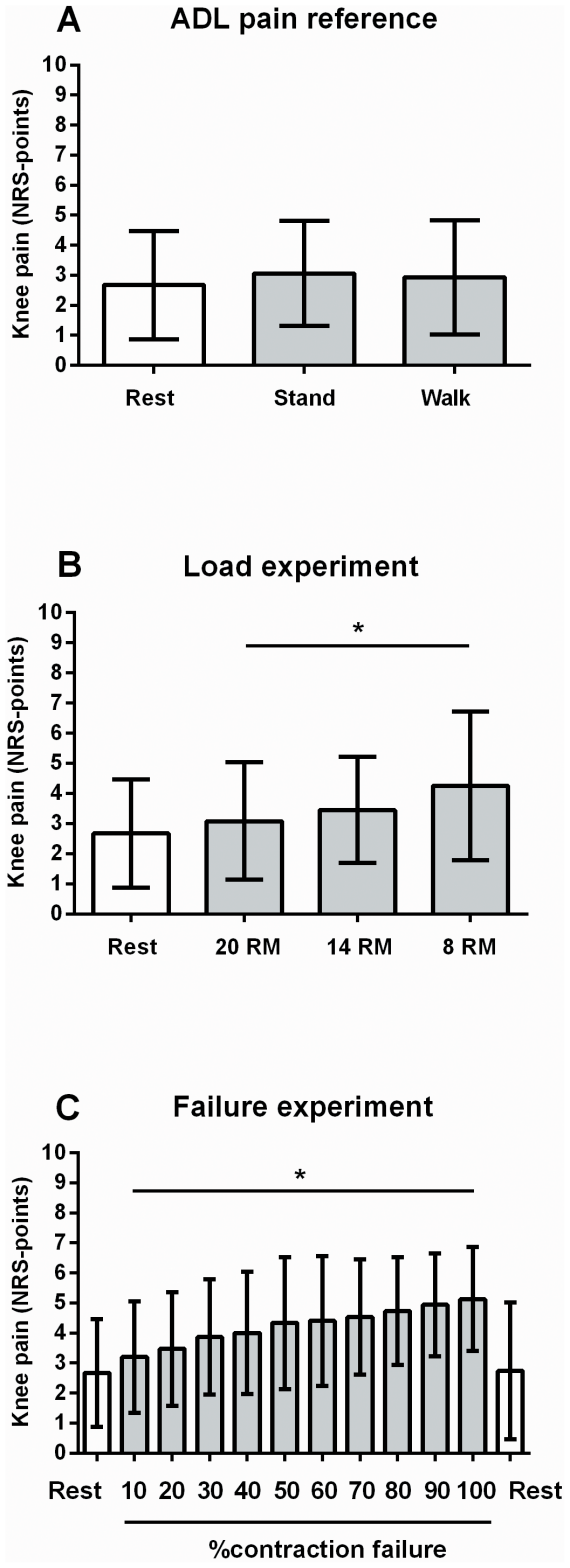
Knee pain in the operated leg during activities of daily living (A, grey bars), during strength training with different loads (B, grey bars), and during strength training until contraction failure (C, grey bars) or at rest (A–C, white bars). Values are means ± 1 SD. * denotes a significant ANOVA main effect of load (B) or repetitions (C) on knee pain during strength training. ADL, activities of daily living; NRS, numerical rating scale; RM, repetition maximum.

Knee pain at rest or during tasks of every-day living was generally low (Figure 2A). A main effect of load on knee pain was observed (P = 0.006), as knee pain increased with increasing loads (Figure 2B). Knee pain reported during the 8 RM load (4.3±2.5 points) was on average 1.2±1.5 points higher than that reported during the 20 RM load (3.1±2.0 points, Figure 2B). Knee pain reported during strength training was comparable to that reported during the tasks of every-day living (Figure 2A).

A main effect of contraction failure on knee pain was also observed (P<0.001), as knee pain increased with repeated contractions (Figure 2C). Knee pain reported during strength training at 100% failure (5.4±1.6 points) was on average 1.9±1.1 points higher than that reported at 10% failure (3.2±1.9 points).

### Indicators of symptom exacerbation (secondary outcomes)

The experimental session did not seem to exacerbate postoperative symptoms, as resting knee pain, knee joint circumference, and knee flexion range of motion did not change over time. Resting knee pain 60 seconds after the final repetition (2.7±2.3 points) was not different from that recorded at rest before strength training (2.7±1.8 points, P = 0.88).

Knee joint circumference 120 seconds after the final repetition (48.7±3.9 cm) was not different from that recorded pre strength training (48.9±4.3 cm, P = 0.88). Knee flexion range of motion 180 seconds after the final repetition (94.9±8.9 cm) was not different from that recorded before strength training (94.9±9.1 cm, P = 0.93).

## Discussion

The main findings of the present study were (i) knee pain increased significantly with increased loading during knee extension strength training shortly following TKA, but the increase was below that defined as clinically relevant, (ii) knee pain increased significantly and clinically relevant with increased muscular fatigue during knee extension strength training shortly following TKA, and (iii) symptom exacerbation due to the experimental protocol was not indicated, as resting knee pain, knee joint circumference and knee flexion range of motion did not change from pre- to post-study.

### Strength training shortly following total knee arthroplasty

Strength training implemented shortly following TKA has been promoted as a rehabilitation modality to enhance postoperative recovery [19], [20], by countering the substantial loss of knee-extension strength after surgery [3]. However, exacerbation of postoperative symptoms is a concern when implementing an intense therapeutic exercise regimen of the knee extensors after TKA, because the knee-extension exercise will also load the operated knee joint. We found that knee pain levels during strength training with loads of 20 to 8 RM (3 to 4 points) were comparable to that reported during every-day tasks in the present study and to that reported during every-day tasks in the first two postoperative weeks after TKA in previous work [21], [22]. From this, it seems possible to load the quadriceps muscle during targeted exercise to a degree, which is generally required for muscle strength and muscle mass gains [10], without increasing pain levels beyond that of every-day living. The reason for this may be that tibiofemoral joint forces, especially compressive forces, are lower during the knee extension exercise, which is an open kinetic chain exercise where the foot in not fixed in space, compared to that during a closed chain exercise, such as the leg press [23], [24].

With respect to the effect of contraction failure on knee pain, we found that knee pain did increase with an average of 1.9±1.1 points to reach 5.4 ± 1.6 points at 100% failure, which was a greater increase than that defined as the minimal clinically important difference. The single set of knee extensions to failure was performed with 14 RM loading, which was the second heaviest load used in the load experiment. The pain level experienced during the 4 non-fatiguing repetitions with the 14 RM load in the load experiment was comparable to that of the 10 to 40% failure also with the 14 RM load (3 to 4 points) in the failure experiment. From 50 to 100% failure, it seemed as if knee pain started to accumulate, possibly indicating acute tissue sensitization to the same mechanical stimulus (14 RM load). Pertaining to this issue, there is no reason to believe that this was caused by painful accumulation of metabolites in the contracting quadriceps muscle, as it was emphasized during the familiarization session as well as during the experimental session that the pain rating should apply to the operated knee joint and not the anterior thigh. When asked, the patients also indicated that it was not difficult to distinguish the two.

The resting pain, knee joint circumference, and knee flexion range of motion recordings made just after the final contraction in the failure experiment indicated that the experimental protocol did not exacerbate postoperative symptoms, as might be expected due to central sensitization of the nociceptive system during exercise. The resting pain, knee circumference, and range of motion recordings made just after the final contraction were no different from that recorded before the set, and comparable to that reported previously [21], [25]. Interestingly, although it seemed as if pain accumulated during the set to failure and reached an average of 5.4 ± 1.6 points at 100% failure, the recording of resting knee pain made only 60 seconds after the final repetition was as low as 2.7 ± 2.3 points on average. So, it seems that strength training the knee extensors in a single set to or near contraction failure with a load of 14 RM is painful – and may be too painful for some patients to perform this early after surgery – but ceasing the set, returns pain levels to that experienced at rest before the set. The first two weeks following discharge seems to be the time period in which patients experience the worst postoperative pain following TKA [26]. So, the prescribing therapist and patient may have to agree on a loading (RM) that induces self-reported pain of no more than 5 points on a numerical ranking scale and subsides shortly after ending the session, according to the pain-monitoring model [27]. This was also the reason for setting the minimal clinically important difference in pain to 1.5 points, given that pain levels of approximately 3.3 points were reported in the pilot study during contractions with the lightest load or least muscular fatigue. As no pain recordings were made beyond 60 seconds after the final repetition in the present study, we do not know anything about pain levels the following time. However, a similar strength training protocol implemented shortly following TKA, and with multiple pain recordings, showed a decrease in knee pain over time, despite an increase in absolute training loads [25].

The present data do not directly support an acute analgesic effect of exercise on postoperative pain after TKA. It does, however, suggest that one bout of exercise in the form of strength training targeting the quadriceps muscle specifically, does not acutely increase postoperative knee pain shortly following TKA. Generally, exercise has been reported to induce analgesic effects in experimental animal and clinical pain models and in some types of chronic pain [28], [29]. With respect to postoperative pain after TKA, it was recently reported that mobilization by standardized walking, reduced knee pain at rest and during walking, while increasing knee pain pressure thresholds at rest [30]. Animal data on the effect of exercise on postoperative pain also point to an analgesic effect. Postoperative exercise in Sprague-Dawley rats reduced sensitivity to von Frey stimuli, as well as reduced the expression of inflammatory cytokines [29].

### Limitations

A few precautions should be taken when interpreting the present results. Firstly, the external validity of the findings may seem limited, due to the sample of 15 patients. However, the study used a within-subject design and was sufficiently powered to answer the primary research question. Secondly, we did not standardize postoperative medication. However, as mentioned previously, the study used a within-subject design, and both the load and failure experiments were performed the same day in less than two hours, which is the time point that the conclusion reflects. It cannot be ruled out that the dose-response relationships determined in the load and failure experiments were not to some extent influenced by differences in the use of postoperative medication or time point after surgery.

## Conclusion

Both loading and repetitions performed to contraction failure during knee extension strength training increased post-operative knee pain recorded during strength training, shortly following TKA. However, only the increase in knee pain induced by repetitions performed to contraction failure exceeded that defined as clinically relevant, and this increase in knee pain was very short-lived.

## Supporting Information

Supporting Information S1 Brief study protocol approved by The Committees on Biomedical Research Ethics for the Capital Region of Denmark (ref: H-4-2011-fsp (108)) in native tongue (A), and a translated version (B). The Committee decided that a full study protocol was not needed for approval, as the study was part of existing clinical practice.
(PDF)

Supporting Information S2 STROBE checklist of items that should be included in reports of cohort studies.
(PDF)
