# Supplementary material for: Knee Pain during Strength Training Shortly following Fast-Track Total Knee Arthroplasty: A Cross-Sectional Study
Source: PLoS One. 2014 Mar 10;9(3):e91107. doi: 10.1371/journal.pone.0091107 (PMC3948740; doi:10.1371/journal.pone.0091107)
Supplement: Supporting Information S1 — Brief study protocol approved by The Committees on Biomedical Research Ethics for the Capital Region of Denmark (ref: H-4-2011-fsp (108)) in native tongue (A), and a translated version (B). The Committee decided that a full study protocol was not needed for approval, as the study was part of existing clinical practice. (PDF) [file pone.0091107.s001.pdf]

(A) Registrering af smerter under genoptræningsøvelser efter indsættelse af total knæledsprotese

Thomas Linding Jakobsen og Thomas Bandholm; Klinisk Forskningscenter, Hvidovre Hospital

INTRODUKTION

Total knæprotese (TKA) tilbydes patienter med alvorlig slidgigt i knæet med henblik på at reducere knæsmerter og øge funktionsevnen (Leopold, 2009). Umiddelbart efter operationen har patienterne et markant fald i knæekstensionsstyrke og funktionsevne (Mizner et al., 2005; Stevens et al., 2003), hvilket betyder, at de er mindre selvhjulpne sammenlignet med deres præ-kirurgiske niveau.

Den gennemsnitlige knæekstensionsstyrke-reduktion efter TKA ser ud til at være henholdsvis 80% ved udskrivelse efter 2.4 postoperative nætter (Holm et al., 2010) og 60% en måned post-operativt (Mizner et al., 2005; Stevens et al., 2003). Idet knæekstensionsstyrke-reduktionen er relateret til forringelsen af funktionsevne efter TKA (Holm et al., 2010) er det ønskeligt at genvinde og gerne øge knæekstensionsstyrken i forhold til det præ-kirurgiske niveau. Det kan eksempelvis gøres via genoptræning, der inkluderer progressiv styrketræning som en delkomponent (Holm & Kehlet, 2009). Forudsætningen for, at denne træningsform kan implementeres umiddelbart efter TKA er, at den tolereres smertemæssigt og smerterne aftager med tiden, selvom træningen stiger i intensitet. Dette har vi for nyligt vist (Jakobsen et al., 2011). Vi mangler dog en lidt mere detaljeret undersøgelse at knæsmerter under udførelse af styrketræning ved forskellige belastninger og gentagelser efter indsættelse af TKA.

FORMÅL

Projektets formål er at beskrive patienternes selvoplevede smerter for de to hyppigst anvendte styrketræningsøvelser for det opererede ben i de første uger efter TKA.

MATERIALE OG METODE

Fireogtyve patienter med nyindsat total knæledsprotese uden belastnings- eller bevægerestriktioner opstarter i den første uge efter operationen standard genoptræning i deres hjem-kommune. De vil i den forbindelse blive bedt om at vurdere deres knæsmerter på en numerisk rangskala fra 0 til 10 under udførelse af styrketræning i øvelserne knæekstension og benpress ved forskellige belastninger og gentagelser (se figur 1). Før og efter hvert træningssæt registreres også knæledsomfang (hævelse) med målebånd og bevægelighed af det opererede knæ med en vinkelmåler (Jakobsen et al., 2009).

PRAKTISKE FORHOLD

Undersøgelsen finder sted som en del af den nuværende genoptræning af patienter med TKA, som den foregår i patienternes hjemkommuner. De kommunerne genoptræningssteder anvender rutinemæssigt styrketræning som en del af genoptræningen af denne patientgruppe og spørger ligeledes rutinemæssigt til smerter og måler knæets bevægelighed i det opererede ben. Det vil rent praktisk sige, at de ovenfor skitserede målinger kun adskiller sig fra nuværende praksis ved, at registreringen bliver lidt mere systematisk.

ETISKE OVERVEJELSER

Det vurderes ikke, at projektet indeholder etiske problemer. Alle inkluderede patienter modtager den normale genoptræning efter total knæledsprotese, som den tilbydes i det kommunale genoptræningsforløb.

HVAD ER DE KLINISKE PERSPEKTIVER?

Denne forventede positive effekt vil kunne udnyttes i den tidlige genoptræning, der følger umiddelbart efter operationen. Styrketræning er vigtigt i forhold til at genvinde tabet af muskelstyrke, hvilket forårsages af operationen. Derfor er det vigtigt at vide, hvilke styrketræningsøvelser, belastninger og gentagelser, som patienterne tolererer bedst (med færrest knæsmerter) tidligt postoperativt.

REFERENCER

Holm B, Kristensen MT, Husted H, Bencke J, Kehlet H, Bandholm T (2010). Loss of knee-extension strength is related to knee swelling after total knee arthroplasty. Arch Phys Med Rehabil 91: 1770-1776.

Holm B, Kehlet H (2009). [Genoptræning efter total knæalloplastik]. Ugeskr Laeger 171: 691-694. Danish.

Jakobsen TL, Christensen M, Christensen SS, Olsen MMI, Bandholm T (2009). Reliability of knee-joint range of motion and circumference measurements after total knee replacement. Physiotherapy Research International 15: 126-134.

Jakobsen, T. L., Husted, Kehlet, H., and Bandholm, T. (2011).Progressive strength training (10 RM) commenced immediately after fast-track total knee arthroplasty: is it feasible? Disabil.Rehabil. (Epub ahead of print).

Leopold SS (2009). Minimally invasive total knee arthroplasty for osteoarthritis. N Engl J Med 360: 1749-1758.

Mizner RL, Petterson SC, Stevens JE, Vandenborne K, Snyder-Mackler L (2005). Early quadriceps strength loss after total knee arthroplasty. The contributions of muscle atrophy and failure of voluntary muscle activation. J Bone Joint Surg Am 87, 1047-1053.

Stevens JE, Mizner RL, Snyder-Mackler L (2003). Quadriceps strength and volitional activation before and after total knee arthroplasty for osteoarthritis. J Orthop Res 21; 775-779

(B) Registration of knee pain during rehabilitation exercises after total knee arthroplasty

Thomas Linding Jakobsen and Thomas Bandholm, Clinical Research Centre, Copenhagen University Hospital, Hvidovre, Denmark.

Introduction

Total knee arthroplasty (TKA) is offered to patients with severe knee osteoarthritis to reduce knee pain and improve functional performance (Leopold, 2009). Shortly following surgery, patients experience a substantial loss of knee-extension strength and functional performance (Mizner et al., 2005; Stevens et al., 2003), making them more dependent postoperatively, as compared to pre-surgery.

The average loss of knee-extension strength after TKA is around 80% at discharge after 2.4 postoperative nights (Holm et al., 2010), and 60% 1 month postoperatively (Mizner et al., 2005; Stevens et al., 2003). As the loss of knee-extension strength is related to the loss of functional performance after TKA (Holm et al 2010), it is desirable to reach the strength level observed preoperatively, or further than that. This is likely achievable through rehabilitation that includes progressive strength training as a rehabilitation modality (Holm & Kehlet, 2009) It requires, however, that such exercise implemented shortly after TKA is tolerable with respect to knee pain, even though the exercise intensity increases progressively. This has recently been indicated (Jakobsen et al., 2011). However, we lack a more detailed description of knee pain while patients perform progressive strength training at different training loads and repetitions after TKA.

Purpose

The project aims to describe patients' self-perceived knee pain during the 2 most commonly used strength training exercises for the operated leg in the first weeks after TKA.

Material and methods

Twenty-four patients, having received a recent primary TKA and with no loading or movement restrictions, are referred to outpatient rehabilitation in their home county approximately 1 week after surgery. During their rehabilitation, they will be asked to rate their knee pain on a numerical ranking scale ranging from 0 to 10, while they perform the strength training exercises: knee-extension and leg press, at different loads and repetitions (see Figure 1). Before and after each strength training set, knee swelling (knee joint circumference) and range of motion of the operated knee joint, will be assessed, using a tape measure and a goniometer, respectively (Jakobsen et al., 2009).

Practical matters

The project is conducted as an integrated part of the outpatient rehabilitation for patients with TKA, as it takes place in patients' home counties. The local rehabilitation sites already use strength training as part of their rehabilitation of these patients. During this, the physiotherapists routinely measure knee pain and knee joint range of motion of the operated leg in patients with TKA. It means that for the present project, the physiotherapists will only have to register knee pain and range of motion slightly more systematically than is already part of routine practise.

Ethical considerations
The project does not appear to be associated with ethical problems. All the included patients will follow routine outpatient practise after TKA, as it is commonly offered at their home county.

What are the clinical perspectives?
The knowledge gathered from this project will have the potential to be implemented in the rehabilitation of patients after TKA, shortly following surgery. As progressive strength training is likely imperative for the regain of knee-extension strength following surgery, it is important to know which exercises, loads and repetitions that are tolerated the best by these patients shortly following surgery.

REFERENCER
Holm B, Kristensen MT, Husted H, Bencke J, Kehlet H, Bandholm T (2010). Loss of knee-extension strength is related to knee swelling after total knee arthroplasty. Arch Phys Med Rehabil 91: 1770-1776.

Holm B, Kehlet H (2009). [Genoptræning efter total knæalloplastik]. Ugeskr Laeger 171: 691-694. Danish.

Jakobsen TL, Christensen M, Christensen SS, Olsen MMI, Bandholm T (2009). Reliability of knee-joint range of motion and circumference measurements after total knee replacement. Physiotherapy Research International 15: 126-134.

Jakobsen, T. L., Husted, Kehlet, H., and Bandholm, T. (2011).Progressive strength training (10 RM) commenced immediately after fast-track total knee arthroplasty: is it feasible? Disabil.Rehabil. (Epub ahead of print).

Leopold SS (2009). Minimally invasive total knee arthroplasty for osteoarthritis. N Engl J Med 360: 1749-1758.

Mizner RL, Petterson SC, Stevens JE, Vandenborne K, Snyder-Mackler L (2005). Early quadriceps strength loss after total knee arthroplasty. The contributions of muscle atrophy and failure of voluntary muscle activation. J Bone Joint Surg Am 87, 1047-1053.

Stevens JE, Mizner RL, Snyder-Mackler L (2003). Quadriceps strength and volitional activation before and after total knee arthroplasty for osteoarthritis. J Orthop Res 21; 775-779
